# Evaluation of the relative roles of the Tabanidae and Glossinidae in the transmission of trypanosomosis in drug resistance hotspots in Mozambique

**DOI:** 10.1186/s13071-020-04087-1

**Published:** 2020-04-29

**Authors:** Fernando C. Mulandane, Louwtjie P. Snyman, Denise R. A. Brito, Jeremy Bouyer, José Fafetine, Jan Van Den Abbeele, Marinda Oosthuizen, Vincent Delespaux, Luis Neves

**Affiliations:** 1 Eduardo Mondlane University, Biotechnology Center (CB-EMU), Maputo, Mozambique; 2 Vectors and Vector Borne Diseases Research Program, Department of Veterinary Tropical Diseases, University of Pretoria, Pretoria, South Africa; 3 Durban Museum of Natural History, Durban, South Africa; 4 CIRAD, UMR ASTRE CIRAD-INRA (Animal, Health, Territories, Risks and Ecosystems), Campus International de Baillarguet, 34398 Montpellier Cedex 05, France; 5 Insect Pest Control Laboratory, Joint Food and Agriculture Organization of the United Nations/International Atomic Energy Agency Programme of Nuclear Techniques in Food and Agriculture, 1400 Vienna, Austria; 6 Department of Biomedical Sciences, Institute of Tropical Medicine Antwerp, Antwerp, Belgium; 7 Bio-engineering Sciences, Vrije Universiteit Brussel, Brussel, Belgium

**Keywords:** African animal trypanosomosis, Hematophagous insects, Tsetse fly, Tabanids, *Trypanosoma congolense*, Transmission

## Abstract

**Background:**

Tsetse flies (Diptera: Glossinidae) and tabanids (Diptera: Tabanidae) are haematophagous insects of medical and veterinary importance due to their respective role in the biological and mechanical transmission of trypanosomes. Few studies on the distribution and relative abundance of both families have been conducted in Mozambique since the country’s independence. Despite Nicoadala, Mozambique, being a multiple trypanocidal drug resistance hotspot no information regarding the distribution, seasonality or infection rates of fly-vectors are available. This is, however, crucial to understanding the epidemiology of trypanosomosis and to refine vector management.

**Methods:**

For 365 days, 55 traps (20 NGU traps, 20 horizontal traps and 15 Epsilon traps) were deployed in three grazing areas of Nicoadala District: Namitangurine (25 traps); Zalala (15 traps); and Botao (15 traps). Flies were collected weekly and preserved in 70% ethanol. Identification using morphological keys was followed by molecular confirmation using cytochrome *c* oxidase subunit 1 gene. Trap efficiency, species distribution and seasonal abundance were also assessed. To determine trypanosome infection rates, DNA was extracted from the captured flies, and submitted to *18S* PCR-RFLP screening for the detection of *Trypanosoma*.

**Results:**

In total, 4379 tabanids (of 10 species) and 24 tsetse flies (of 3 species), were caught. NGU traps were more effective in capturing both the Tabanidae and Glossinidae. Higher abundance and species diversity were observed in Namitangurine followed by Zalala and Botao. Tabanid abundance was approximately double during the rainy season compared to the dry season. *Trypanosoma congolense* and *T. theileri* were detected in the flies with overall infection rates of 75% for tsetse flies and 13% for tabanids. *Atylotus agrestis* had the highest infection rate of the tabanid species. The only pathogenic trypanosome detected was *T. congolense*.

**Conclusions:**

Despite the low numbers of tsetse flies captured, it can be assumed that they are still the cyclical vectors of trypanosomosis in the area. However, the high numbers of tabanids captured, associated to their demonstrated capacity of transmitting trypanosomes mechanically, suggest an important role in the epidemiology of trypanosomosis in the Nicoadala district. These results on the composition of tsetse and tabanid populations as well as the observed infection rates, should be considered when defining strategies to control the disease.
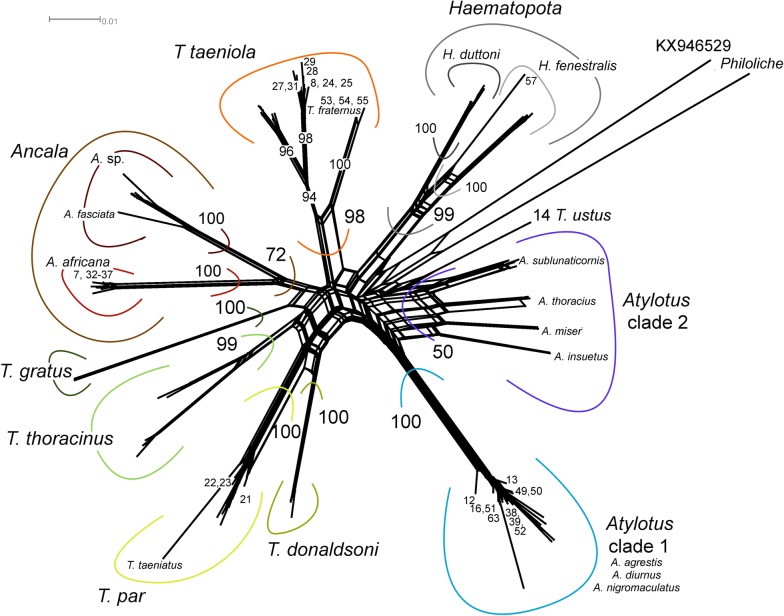

## Background

Tsetse-transmitted animal African trypanosomosis is one the most important diseases in sub-Saharan Africa [1] where it causes the death of thousands of heads of cattle per year and reduces the availability of meat, milk and other cattle-derived products [2–5]. Additionally, fertile areas are often unsuitable for cattle keeping due to the presence of tsetse flies, the biological vector of the pathogen [6, 7]. The presence of tabanid flies, mechanical vectors of many human and veterinary disease-causing agents, including *Trypanosoma* species, the cause of animal African trypanosomosis, intensifies the problem [8, 9].

Tsetse flies (Diptera: Glossinidae), are thought to comprise 34 species and subspecies from a single genus, *Glossina* [10–13]. *Glossina* spp. males and females feed exclusively on blood, which is required for all metabolic processes, and are subsequently ideal pathogen vectors. In Mozambique, *G. morsitans*, *G. pallidipes*, *G. brevipalpis* and *G. austeni* have all been recorded [14–16]. Species of the Tabanidae, in contrast, need nectar for the metabolic processes, with only females of most species requiring blood for the production of eggs [8, 9]. Approximately 4400 species have been described, currently divided into the subfamilies Pangoniinae, Tabaninae and Chrysopsinae; however, the higher classification remains unsettled [9, 17, 18]. Tabanids have been incriminated as vectors of livestock pathogens, including trypanosomes, and are considered one of the most efficient mechanical vectors due to their high mobility, interrupted feeding and large mouthparts [19–21].

Scarce information regarding the distribution of tsetse flies and tabanids in Mozambique, particularly in the Nicoadala District, an area identified as trypanocidal resistance hotspot, through a block treatment study [22], is currently available. Furthermore, their role in the transmission of trypanosomes requires further elucidation, especially in the Afrotropics [23]. Moreover, little has been done to update the knowledge on the distribution and composition of hematophagous fly populations in Mozambique since the studies of Dias [24] and Oldroyd [25–27]. The lack of the aforementioned information, together with constant reports of high trypanosomosis prevalence in Nicoadala, urge for an accurate monitoring study targeting vectors of trypanosomosis and is the aim of this study. This study would allow an updated view on the respective vector species composition, with the ultimate goal of gaining a better understanding of the role said vectors play in maintaining and spreading trypanosomes in the trypanocidal drug resistance foci of Nicoadala District. Such results are of paramount importance for implementing regional vector control and/or management as well as subsequent policy making.

The use of trapping to determine the distribution of hematophagous insects have been extensively described in the scientific literature [28–33]. Currently, a plethora of different traps are available for collecting hematophagous brachyceran flies. Here, the horizontal or H trap, developed in South Africa for *G. brevipalpis* and *G. austeni* [34], the Epsilon trap, developed in Zimbabwe as an alternative trap for savanna species such as *G. pallidipes* and *G. morsitans* [35] and the NGU trap, developed in Kenya for catching savannah flies such as *G. pallidipes* and also effective for tabanids [36], were selected given the species present in Mozambique.

The *18S* PCR-RFLP method is effective for detecting *Trypanosoma*, or mixed parasite infections, from fresh or ethanol-preserved flies. The method has been shown to be accurate for identification of parasites up to the subspecies level [37, 38].

This study aims to assess the species composition and abundance of the Tabanidae and Glossinidae in three foci within Nicoadala District, Mozambique. Additionally, the study aims to report on *Trypanosoma* infection rates of said vectors, trapping efficacy and cytochrome *c* oxidase subunit 1 (*cox*1) gene as a species delimitation tool.

## Methods

### Study area

Three grazing areas, Botao, Namitangurine and Zalala, all located in the Nicoadala District (17.608°E, 36.820°N), were used for the survey. The region has a rainy tropical savannah climate according to the Köppen-Geiger system [39, 40], with two seasons: rainy and dry. The average temperature in Nicoadala is 25.6 °C with about 1428 mm annual rainfall. In Zalala, the vegetation is mostly composed of coconut trees, grasses and small shrubs covering approximately 420 ha. Botao (*c.*1300 ha) is an open forest with large grassland areas. Namitangurine (*c*.1600 ha) is a slightly closed canopy forest with a high diversity of trees and shrubs. The dominant tree species in the district are *Pterocarpus angolensis*, *Swartzia madagascariensis*, *Afzelia quanzensis*, *Millettia stuhlmannii*, *Khaya nyasica*, *Pericopsis angolensis*, *Combretum imberbe*, *Brachystegia spiciformis* and *Pteleopsis myrtifolia.* Both Botao and Namitangurine are areas with pasture and water availability with some wild animals present [41]. Recently, Nicoadala was identified as a multiple trypanocidal drug resistance hotspot, in a block treatment study where trypanosomes in cattle were tested to both isometamidium chloride and diminazene aceturate [22]. As there are no recent studies elucidating the epidemiology of trypanosomosis in the region, it is of crucial importance to develop a study to identify possible vectors of the disease. In addition, this study will help in the understanding of the role of mechanical transmission of the disease in the presence of resistant isolates.

### Sampling

**Fig. 1:**
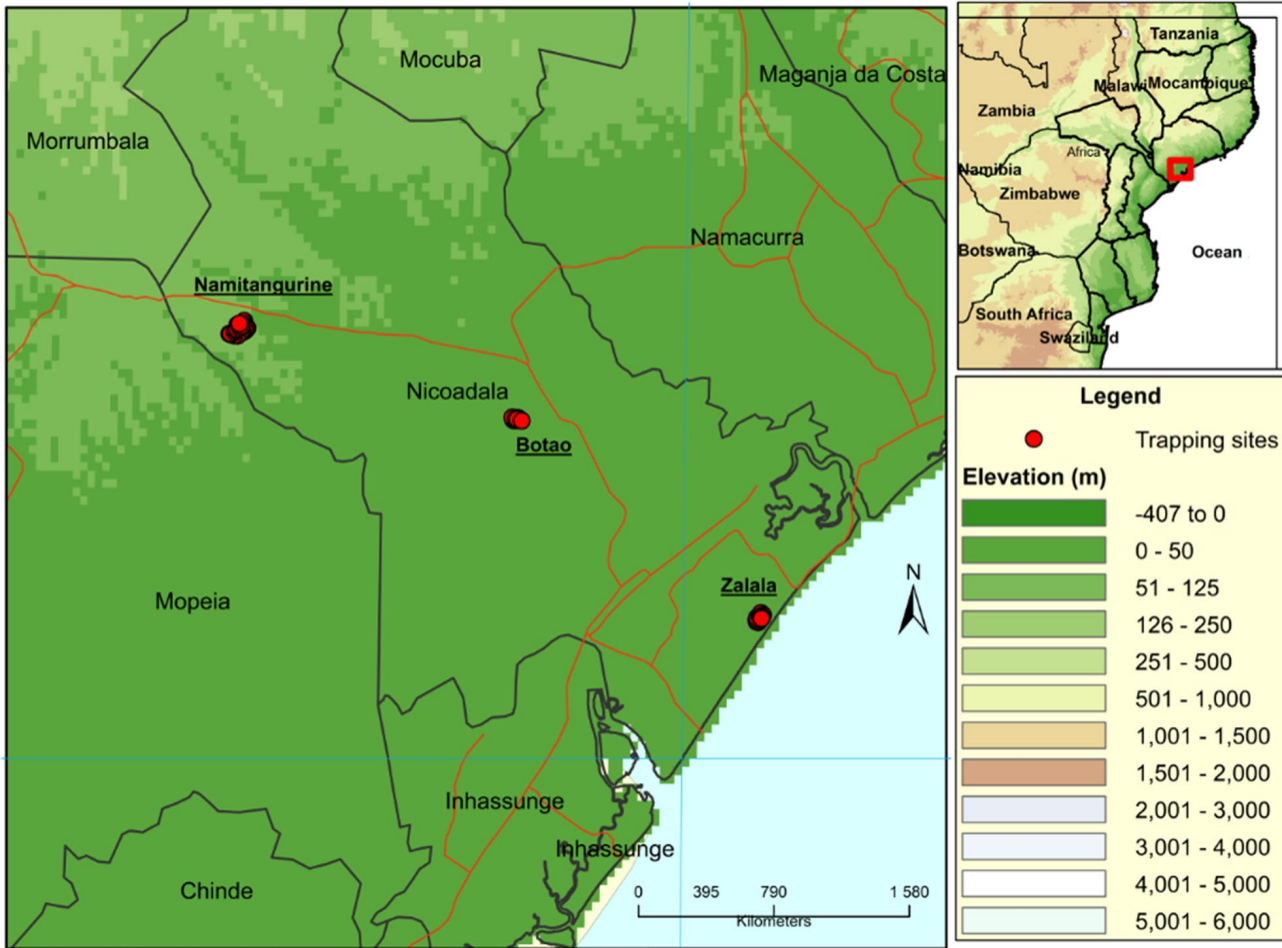
Trapping sites in the three grazing areas in Nicoadala District, central province of Zambezia. The study area is identified by the red square in the map of Mozambique

For 12 months, a total of 5 H traps, 5 Epsilon traps and 5 NGU traps were deployed in Botao, an equal number of traps were deployed in Zalala, and 10 H traps, 5 Epsilon traps and 10 NGU traps were deployed in Namitangurine. Trap numbers were defined by the size of the sampling areas and all traps were placed at least 200 m apart to prevent interference [33, 42]. Trapping sites were georeferenced using a Garmin-GPSMAP®76 (Garmin Ltd., Kansas, USA) (Fig. 1). The traps were left at the deployment sites for the duration of the study [43, 44]. Each trap was baited with 4 polyethylene sachets (150 µm thick, surface area of 30 cm^2^) containing 4 ml of 1-octen-3-ol and one 15 ml plastic bottle with a 5 mm hole on top, containing acetone. Flies were collected weekly and those captured were placed in 1.5 ml tubes containing 1 ml of 70% ethanol until further analysis [16, 45]. All the bottles containing trapped flies were labeled with location, trap number, date and time and then replaced with empty ones.

### Identification of hematophagous insects

All tsetse flies captured were identified using the tsetse control personnel training manual key for *Glossina* species identification, through the analysis of the fly’s morphological traits [11, 46]. Tabanids were identified to the genus and species level using the morphological keys of Oldroyd [25–27] supplemented by Taioe et al. [23] and Dias [24]. To conduct the molecular species delimitation, DNA was extracted using the Chelex® protocol modified from Walsh et al. [47] and Ravel et al. [48]. Polymerase chain reaction (PCR) targeting the cytochrome *c* oxidase subunit 1 (*cox*1) gene was conducted using an Applied Biosystems® thermal cycler (Thermo Fisher Scientific, Göteborg, Sweden). Reactions were performed in a total volume of 20 µl containing, 10 µl of 2× Phusion Flash Master Mix, 0.5 µM of each primer and 6.5 µl of double distilled water (ddH_2_O). PCR conditions were as follows: 98 °C for 10 s, 30 cycles of 98 °C for 1 s, 50 °C for 5 s and 72 °C for 15 s, followed by a final elongation step of 72 °C for 1 min. The primers used were LCO1490 (5′-GGT CAA CAA ATC ATA AAG ATA TTG G-3′) and HCO2198 (5′-TAA ACT TCA GGG TGA CCA AAA AAT CA-3′) and the expected size of the fragment was approximately 653 bp [23, 49]. Genomic DNA from *Glossina brevipalpis* and *G. austeni* (from the Biotechnology Center, Eduardo Mondlane University reference collection) were used as a positive control and ddH_2_O as a negative control. For PCR product visualization, the samples were analysed on 2% agarose gels, where 2 µl of loading dye was mixed with 5 µl of PCR product and loaded onto the gel. A 1 Kb DNA ladder was also loaded (4 µl) for fragment size determination; gel electrophoresis was performed for 45 min at 100 volts. The gels were stained with GelRed (Biotium, Inc., Fremont, CA, USA) using 4 µl per 100 ml of agarose solution directly added before polymerization. Four replicates of the PCR amplicons from the *cox*1 PCR amplification were produced and pooled. After confirmation on agarose gel, 50 µl of the product was sent for sequencing at Inqaba Biotec™ (Pretoria, South Africa).

### Phylogenetic analysis

With the aim of verifying monophyly of the species using molecular methods, and subsequently, infer species identification, all retrieved sequences were viewed and assembled using BioEdit version 7.0.9 [50]. Assembled sequences from species of both the Tabanidae and Glossinidae were separately aligned with various sequences from the GenBank database using the online version of MAFFT with default parameters [51]. The aligned matrices were viewed, edited and truncated in MEGA 7 [52] and used for all analysis. Data-display networks (DDN) were constructed in SplitsTree version 4 [53], from uncorrected p-distances using both parsimony-informative and -uninformative characters. Bootstrap (bs) support for the DDN was calculated from 1000 replicates. jModelTest version 2.1.10, *via* the Cipres Science gateway [54], was used for model estimation. A maximum likelihood (ML) analysis was performed in RAxML version 8 using the estimated models (GTR + G + I). The auto MRE function was invoked for the calculation of bootstrap support [55]. A Bayesian approach for phylogenetic inference (BI) was conducted in MrBayes version 3 [56]. Four simultaneous cold Monte-Carlo Markov Chains searched for 10 million generations, with each 1000th tree sampled. In both cases, TIM1 + G + I estimated using the jModelTest was employed as the substitution model. Before tree construction, the first 15% of the trees were discarded as ‘burn-in’ for the subsequent calculation of the posterior probabilities (pp). Effective sample sizes were calculated and viewed using Tracer version 1.6 [57] where values of > 200 were considered sufficient.

### Glossinidae and Tabanidae diversity and seasonal abundance and trap efficiency

To access species richness and evenness from the grazing areas, alpha (α) diversity was calculated using the Shannon index [58, 59], respectively. Data was processed with Vegan 2.5-2 in R [60]. A comparison of trap efficiency was performed considering both the total number of individuals and the number of species captured by each type of trap. Seasonal abundance was assessed and compared.

### Detection of trypanosome infection rates

A total of 480 (160 specimens from each site) tabanids and all tsetse captured (24 in total) were screened. To estimate the sample size and get a statistically representative number of 160 individuals to be sampled per area, the formula from Cannon & Roe [61] was used. The number was determined when the expected prevalence was set at 10% with a 5% confidence interval and a 5% desired absolute precision. These were selected equally into the four most captured species namely *Tabanus par*, *T. taeniola*, *Atylotus agrestis* and *Ancala africana*. The 24 tsetse flies comprised 17 *Glossina* specimens from Namitangurine (11 *G. brevipalpis*, 4 *G. morsitans* and 2 *G. pallidipes*), 6 *G. brevipalpis* from Botao and 1 *G. brevipalpis* from Zalala. DNA was extracted from individual flies using the ammonium acetate precipitation protocol modified from Bruford et al. [62]. For the molecular detection of trypanosomes in the flies, semi-nested rRNA PCRs were run targeting a fragment of the *18S* ribosomal RNA gene. PCR conditions and gel visualization followed the description by Mulandane et al. [22] and Geysen et al. [38].

### Statistical analysis

Mean infection rate per species, per species/area and per total tabanids/area were calculated. To determine whether observed differences in tsetse and tabanids infection rates, infection rates per area and per species, tabanids distribution, trap efficiency and seasonal abundance were statistically significant, ANOVA with Tukey’s HSD *post-hoc* test was used; Studentʼs t-test was used to determine if significant differences were observed between the seasons. All analyses were carried out with Statistica version 13.3 [63].

## Results

Over a period of 12 months using 55 traps, a total of 4379 tabanids and 24 tsetse flies were captured in three areas of Nicoadala District of which 35.9% in Zalala, 33.4% in Namitangurine and 30.7% in Botao.

Based upon morphological characterization, the captured tabanids belonged to four genera: *Tabanus*, *Ancala*, *Atylotus* and *Haematopota*. The genus *Tabanus* accounted for 73.2% of the total capture (*n* = 3207), followed by the genera *Atylotus* (23.2%; *n* = 1017), *Ancala* (2.4%; *n* = 149) and *Hematopota* (0.14%; *n* = 6). *Tabanus par* and *A. agrestis* were the most frequently caught species (66.8% and 23.2% of the captured flies, respectively). *Tabanus par*, *T. taeniola*, *A. agrestis* and *Ancala africana* were captured in all the three habitats (Table 1). From the 4379 tabanids captured, 16 *Tabanus* specimens were damaged and/or discolored by alcohol that made the morphological identification to the species level unreliable.

**Table 1:** Total flies captured and tested in all three grazing areas, in Nicoadala District

Fly species	Grazing area			Total flies captured
	Zalala	Botao	Namitangurine	
Tabanids	1571	1345	1463	4379
* Tabanus par*	894	1130	900	2924
* Atylotus agrestis*	482	89	446	1017
* Tabanus taeniola*	79	71	92	242
* Ancala africana*	96	47	6	149
* Tabanus gratus*	5	0	7	12
* Tabanus biguttatus*	0	0	1	1
* Tabanus fraternus*	0	2	6	8
* Tabanus denshamii*	1	0	1	2
* Haematopota* spp.	3	3	0	6
* Tabanus ustus*	2	0	0	2
Undetermined	9	3	4	16
Tsetse flies	1	6	15	24
* Glossina brevipalpis*	1	6	11	18
* Glossina m. morsitans*	0	0	4	4
* Glossina pallidipes*	0	0	2	2
Total	1572	1351	1480	4403

Three of the four *Glossina* species present in Mozambique were captured. These species were *G. brevipalpis* (*n* = 18), *G. pallidipes* (*n* = 2) and *G. morsitans morsitans* (*n* = 4). In Botao (*n* = 6) and Zalala (*n* = 1), only *G. brevipalpis* was captured. Namitangurine contributed with more than 50% of the total captured *Glossina* (11 *G. brevipalpis*, 4 *G. morsitans morsitans* and 2 *G. pallidipes*), and thus, the only area where all three *Glossina* species were captured.

**Fig. 2:**
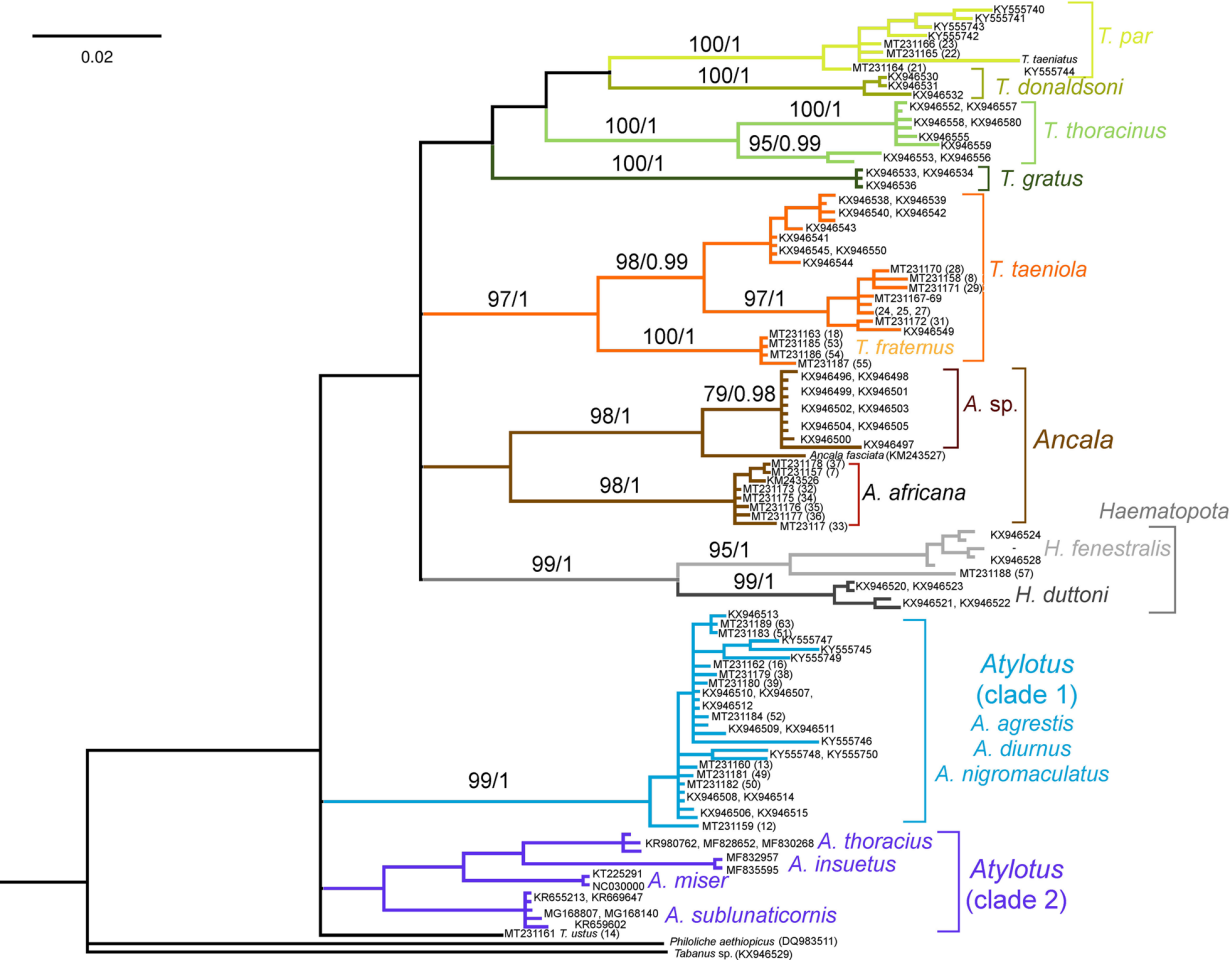
A consensus phylogram constructed in a Bayesian inference analysis using the Tabanidae *cox*1 data. Nodal support presented on the branches are bootstraps (bs) calculated a RAxML analysis (autoMRE function) and posterior probabilities (pp) calculated from a MrBayes analysis. Only bs values > 75 and pp values > 0.95 are shown (bs/pp)

**Fig. 3:**
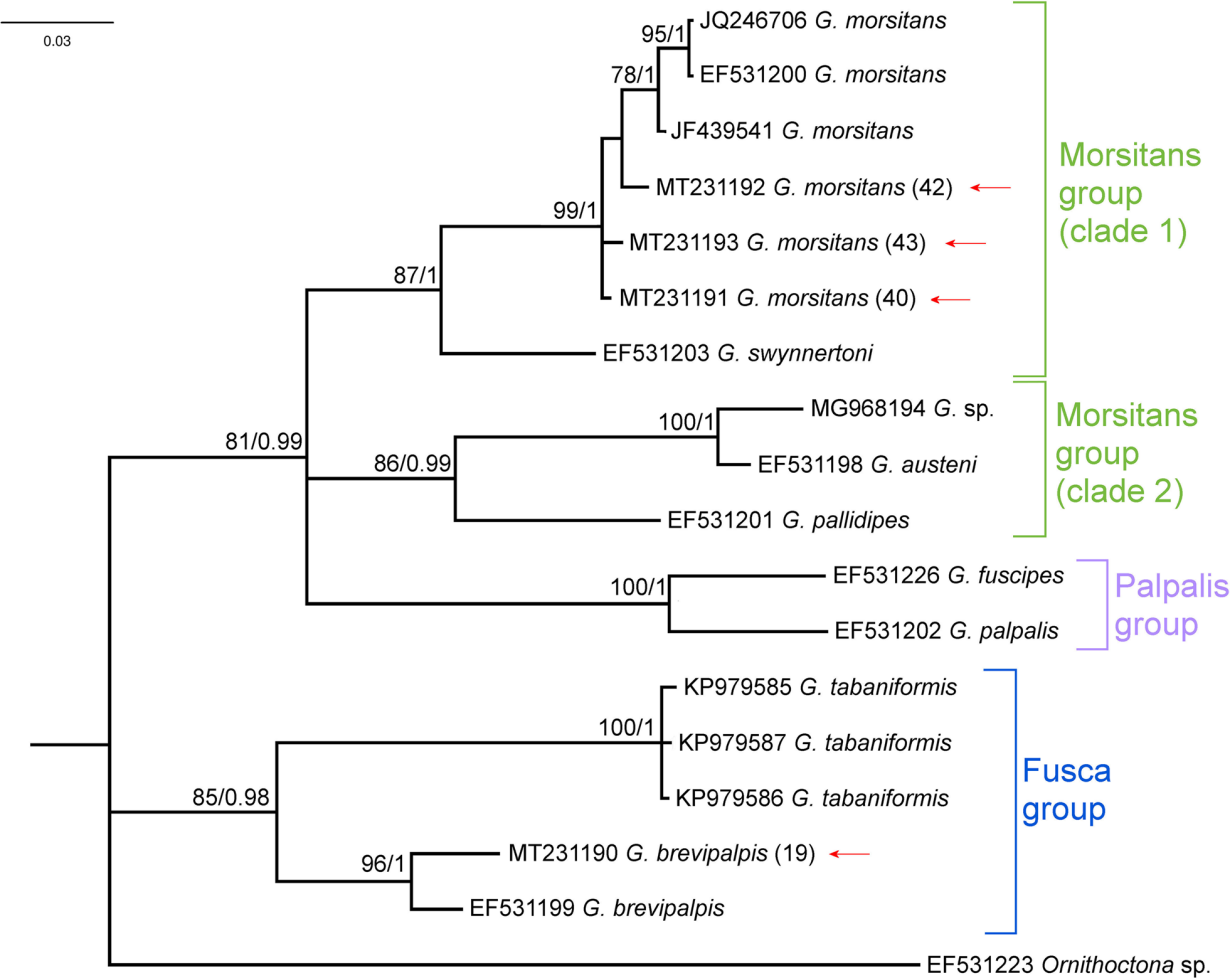
A consensus phylogram recovered from a MrBayes analysis using the Glossinidae *cox*1 data. Nodal support presented on the branches are bootstraps (bs) calculated from a RAxML analysis (autoMRE function) and posterior probabilities (pp) calculated from a MrBayes analysis. Only bs values > 75 and pp values > 0.95 are shown (bs/pp). Red arrows indicate the specimen sequences from this study

The dimensions of the final alignment matrices were 113 taxa and 658 characters and 18 taxa and 659 characters for the Tabanidae and Glossinidae, respectively. Identical substitution models, GTR + G + I (AIC criterion) and TIM1 + G + I (BIC criterion) were recovered for both datasets. Congruent topologies across all methods were recovered (Figs. 2, 3). Three specimens could be confidently identified as *Ancala africana* (nos. 7, 32, 37: DDN bs = 100; ML bs = 98; BI pp = 1) (Fig. 2, Additional file 1: Figure S1). Two *Atylotus* clades were recovered across all methods. All ten specimens sequenced for this study fell within a collapsed node representing *A. agrestis*, *A. diurnus* and *A. nigromaculatus* (DDN bs = 100; ML bs = 99; BI pp = 1) (Fig. 2, Additional file 1: Figure S1). Three specimens (nos. 21–23) could confidently be identified as *T. par* (DDN bs = 100; ML bs = 100; BI pp = 1) (Fig. 2, Additional file 1: Figure S1). An unverified sequence (GenBank: KY555744) submitted by Mugasa et al. [64], as *T. taeniatus* consistently fell within the *T. par* group. Seven specimens (nos. 8, 24, 25, 27–29 and 31) were confidently recovered as *T. taeniola* (DDN bs = 94; ML bs = 98; BI pp = 0.99), and four specimens were identified as *T. fraternus* (nos. 18, 53–55) (DDN bs = 100; ML bs = 100; BI pp = 1) (Fig. 2, Additional file 1: Figure S1). Together, *T. taeniola* and *T. fraternus* formed a well-supported monophyletic clade (DDN bs = 98; ML bs = 97; BI pp = 1). A single specimen (57), formed a well-supported clade with *Haematopota fenestralis* (DDN bs = 100; ML bs = 95; BI pp = 1) (Fig. 2, Additional file 1: Figure S1). The specimen morphologically identified as *T. ustus* (no. 14) could not be verified as belonging to any species-group due to a lack of reference sequences on GenBank.

Congruent topologies, analyzing the Glossinidae *cox*1 data, from all three analyses were recovered (Fig. 3, Additional file 2: Figure S2). In all analyses, the Fusca and Palpalis groups were well-supported monophyletic clades (DDN bs = 99; ML bs = 85; BI pp = 0.98 and DDN bs = 100; ML bs = 100; BI pp = 1, respectively). Two well-supported monophyletic clades representing the Morsitans group were consistently found across all analyses (Clade 1: DDN bs = 94; ML bs = 87; BI pp = 1; and Clade 2: DDN bs = 100; ML bs = 86; BI pp = 0.99) (Fig. 3, Additional file 2: Figure S2). All four specimens could be confidently identified. Three specimens (40, 42 and 43) were molecularly identified as *G. morsitans* (DDN bs = 100; ML bs = 99; BI pp = 1) and one specimen was identified as *G. brevipalpis* (DDN bs = 100; ML bs = 96; BI pp = 1) (Fig. 3, Additional file 2: Figure S2). No sequence data could be generated from the *G. pallidipes* specimen sampled in this study; however, morphological identification was assigned with a high degree of confidence. Metadata for all specimens analysed are provided in Additional file 3: Table S1.

Alpha diversity analysis revealed a slightly higher tabanid species richness and evenness in Zalala (Shannonʼs index of 1.08) and Namitangurine (Shannonʼs index 0.98), with 9 species of tabanids captured in each area. Botao with a Shannonʼs index of 0.64, revealed a lower diversity and evenness in the area.

**Fig. 4:**
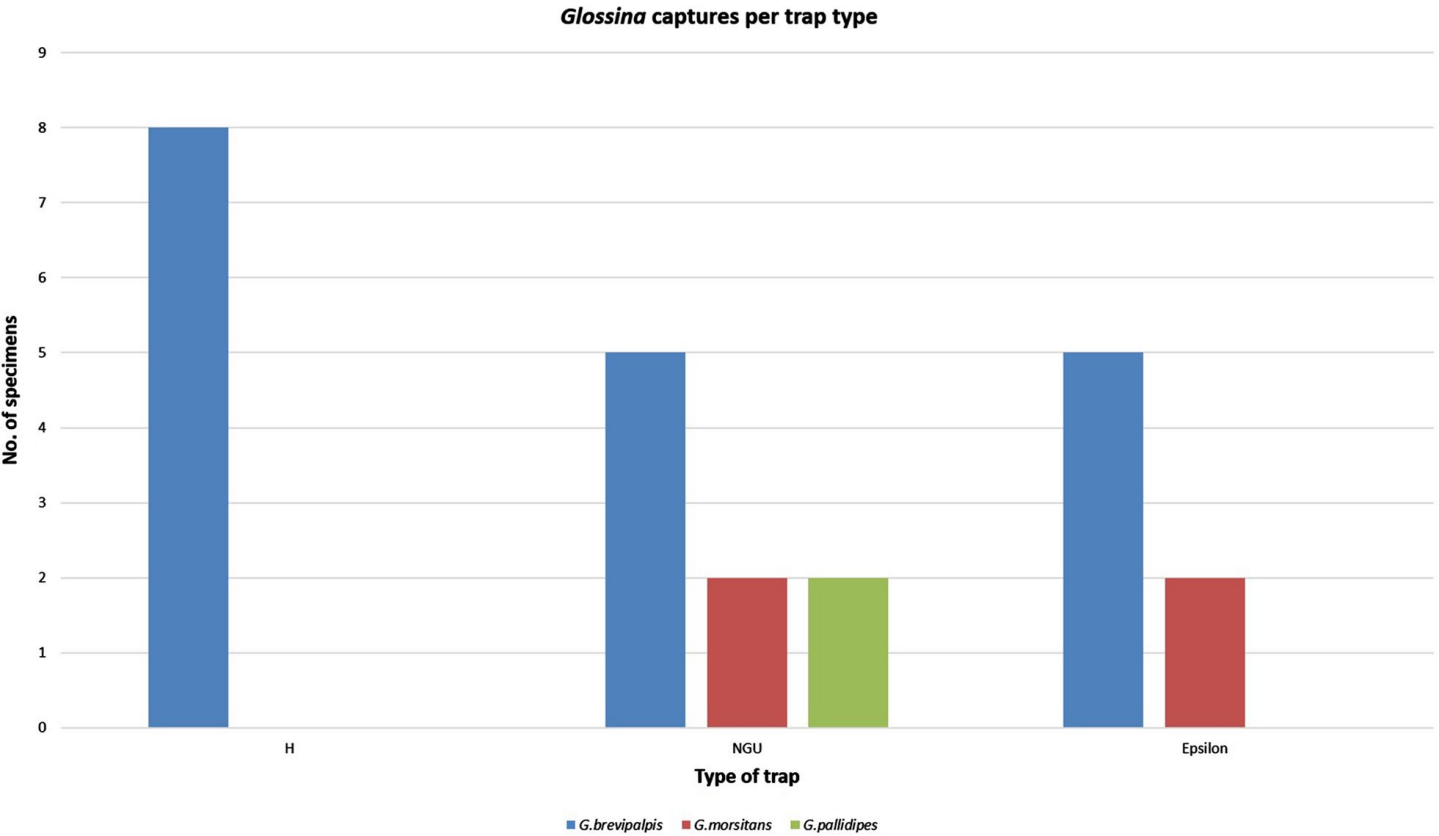
Bar graph showing the total *Glossina* spp. captured by each type of trap

**Fig. 5:**
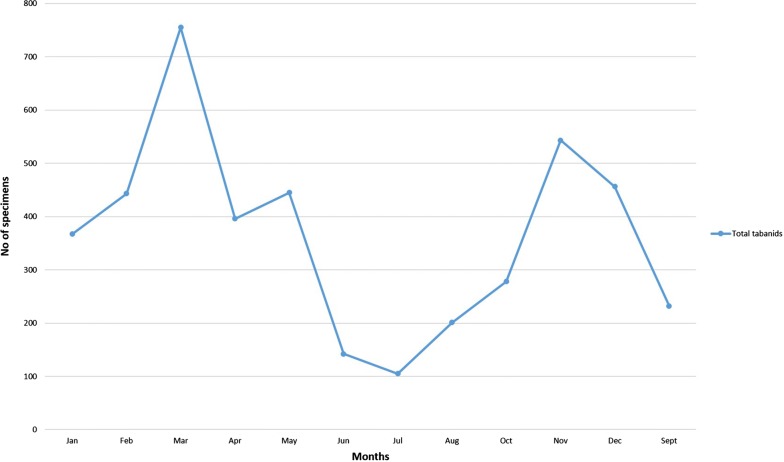
Total tabanids caught during the twelve months of trapping

Analyzing the trap efficiency, NGU (1735 tabanids and 9 tsetse flies captured) demonstrated better performance in general. This trap also successfully captured all three species of *Glossina.* The H traps only captured *G. brevipalpis* while Epsilon traps managed to capture the two tsetse savannah species present in the country (Fig. 4). There was a significant difference (Studentʼs t-test, *t* = 7.44, *df* = 658, *P* < 0.001) between the captures in dry and rainy seasons, with the latter accounting for 70.5% of the total captures. Tabanids had a high activity peak in March (755 tabanids captured) as compared to July (106 tabanids), during which the lowest density was recorded (Fig. 5).

**Fig. 6:**
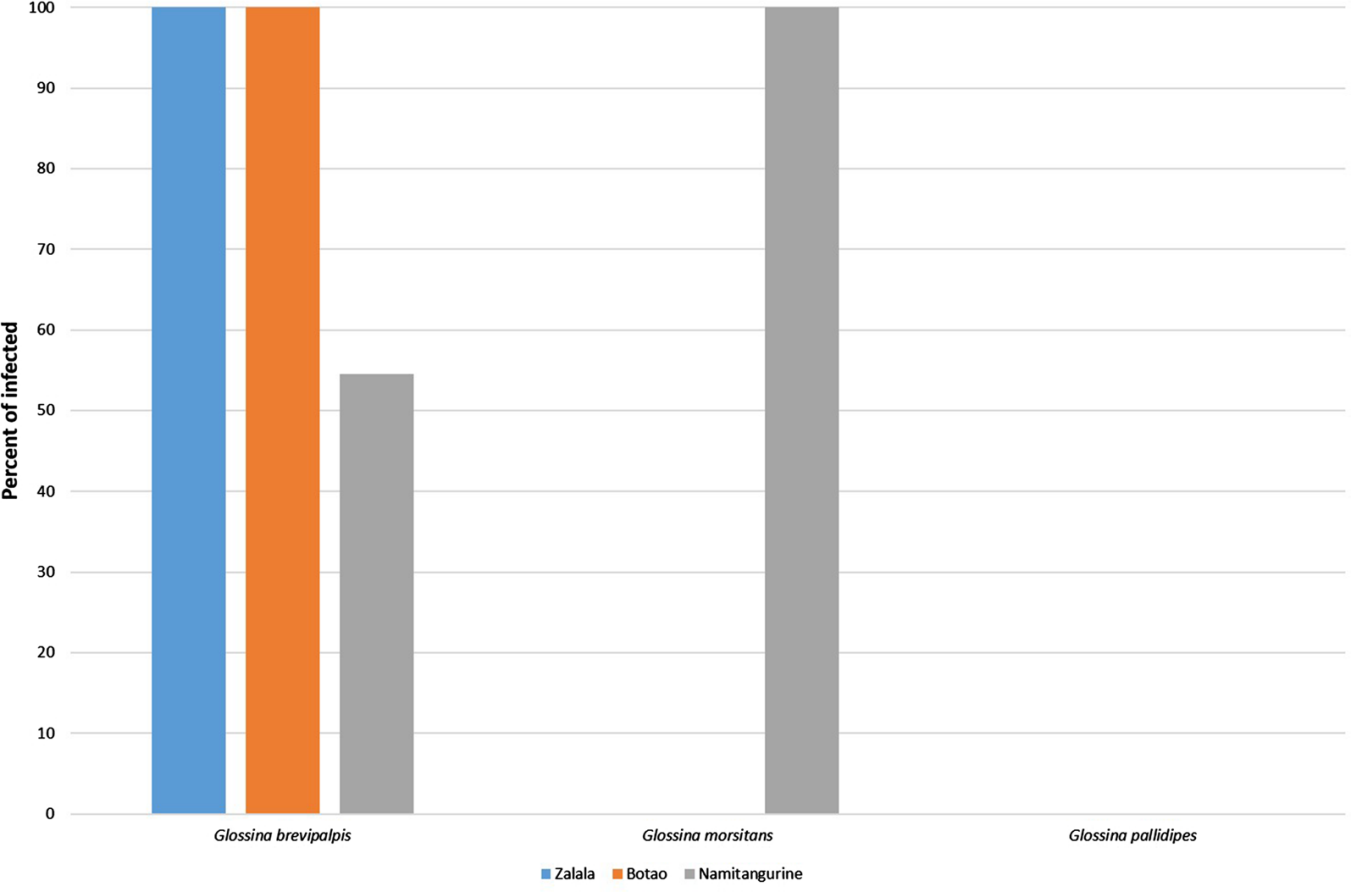
Percentage of infected tsetse flies (total *n* = 24) per sampling area detected using *18S* PCR-RFLP

From the 480 tabanids screened, 13.0% (66/480) tested positive for the presence of *Trypanosoma* spp. The overall trypanosome infection rate for tsetse flies was 75.0% (18/24). Only *T. congolense* infections were detected in *Glossina* spp. and only in *G. brevipalpis* and *G. morsitans* (Fig. 6). Namitangurine with 18.8% (30/160) presented the highest tabanid infection rate followed by Botao with 13.1% (21/160) and Zalala with 9.4% (15/160). There were no significant differences when comparing the infection rates of the tabanids between the three sites (ANOVA: *F*_(2, 9)_ = 0.44, *P* = 0.66).

**Fig. 7:**
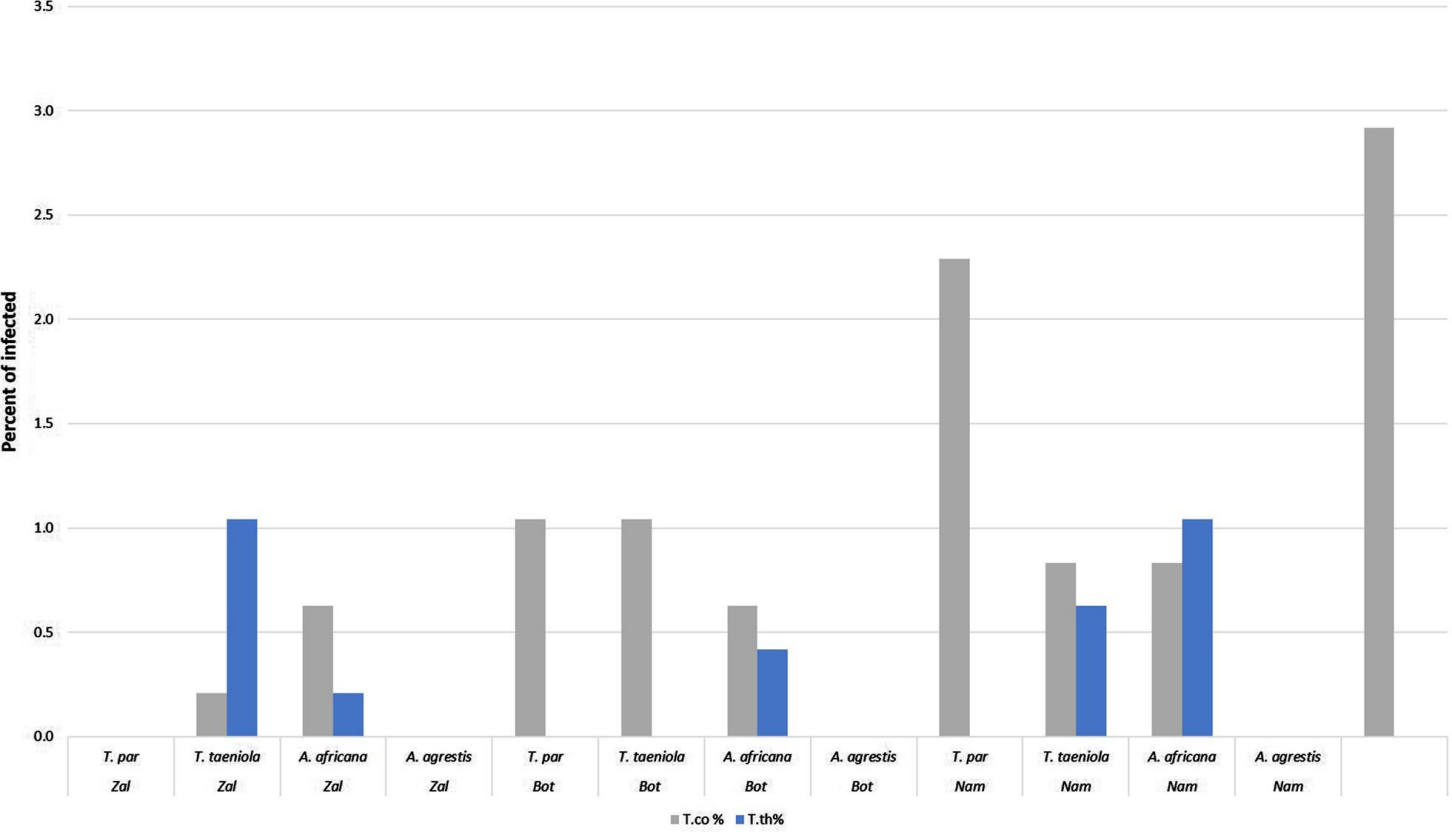
Percentage of infected tabanid flies per species/area and per species of *Trypanosoma* (*n* = 480) as detected by *18S* PCR-RFLP. *Abbreviations*: *T. par*, *Tabanus par*; *T. taeniola*, *Tabanus taeniola*; *A. africana*, *Ancala africana*; *A. agrestis*, *Atylotus agrestis*; T. co, *Trypanosoma congolense*; T. th, *Trypanosoma theileri*; Zal, Zalala; Bot, Botao; Nam, Namitangurine

**Fig. 8:**
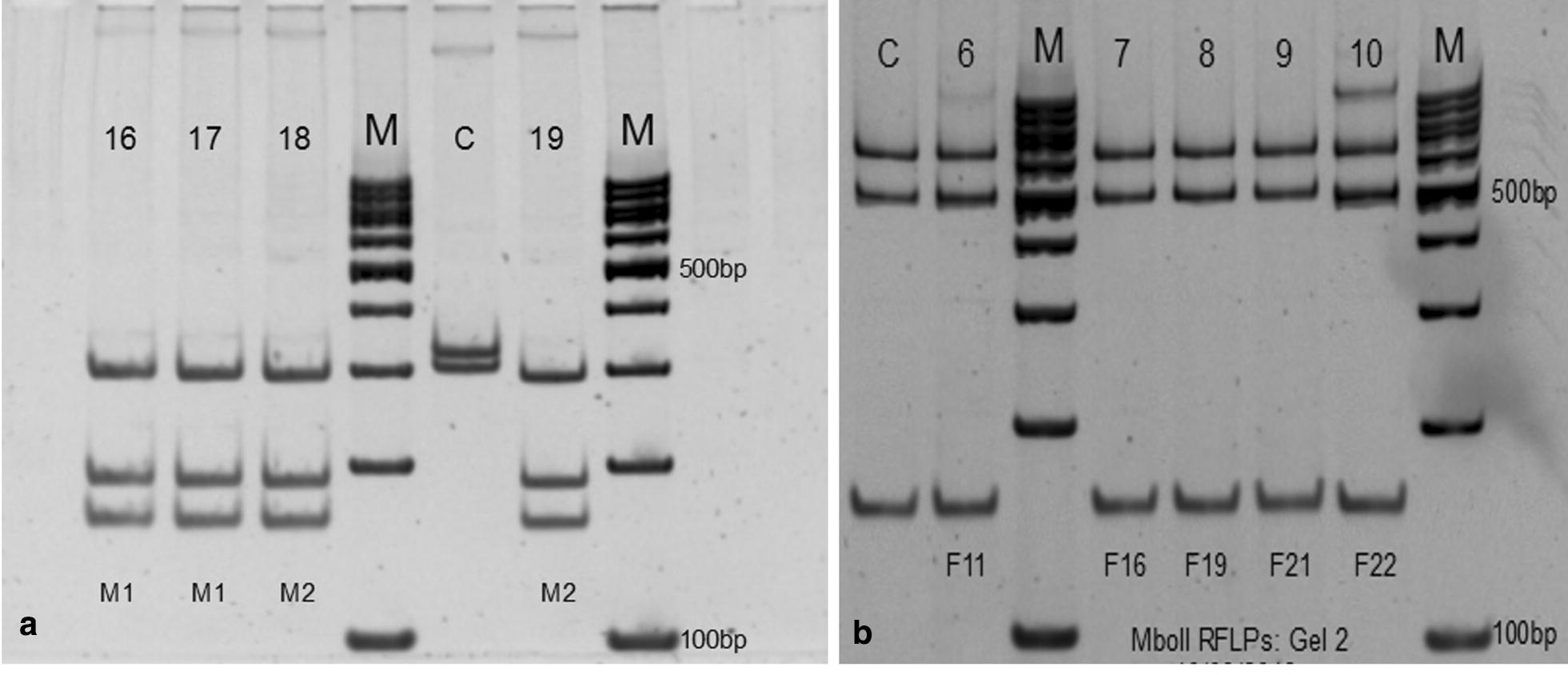
RFLP restriction enzyme analysis using *Msp*I and *Eco*571 (**a**) and *Mbo*II (**b**) digestion of 18 *SSU*-rDNA from *Trypanosoma congolense* (Lanes 16, 18, 9) and *T. theileri* (Lanes 6, 7, 8, 9 and 10) in polyacrylamide gel. Positive control for *T. congolense* (Lane 17) and for *T. theileri* (Lanes C) and a 100-bp DNA ladder (Lanes M) were included on the gels. *Abbreviations*: M1, samples from the first DNA extraction group; M2, samples from the second DNA extraction group; M1/Lane 16, *Glossina brevipalpis*; M1/Lane 18, *Atylotus agrestis*; M2/Lane 19, *Glossina brevipalpis*; F11, fly 11 (*Tabanus par*); F16, fly 16 (*Atylotus agrestis*); F19, fly 19 (*Tabanus par*); F21, fly 21 (*Tabanus taeniola*); F22, fly 22 (*Atylotus agrestis*)

Species comparisons revealed that *A. agrestis* had the highest trypanosome infection rate with 6.25% (30/480), followed by *T. par* and *T. taeniola*, both yielding 3.8% of infected flies (18/480). No *A. africana* specimen tested positive for trypanosome infection. There was significant difference in infection rate between the three species where *Trypanosoma* infections were detected (ANOVA, *F*_(3, 8)_ = 7.84, *P* = 0.009, *post-hoc* test: Tukey’s HSD, *P* = 1.0 for *T. par vs T. taeniola*, *P* = 0.19 for *T. par vs A. agrestis*, *P* = 0.23 for *A. agrestis vs T. taeniola* and *P* = 0.01 for *A. agrestis vs A. africana*). However, the *post-hoc* test only showed significant difference between *A. agrestis* and *A. africana*, which represents the difference between the species with the highest infection rate and the species with no infection. Comparing the infection rate per species/area, *A. agrestis* remained the species with the highest infection rate in both Namitangurine and Botao. In Zalala, *T. par* had a higher infection rate (Fig. 7). The trypanosome DNA detected in tabanids belonged to *T. congolense* (the only pathogenic trypanosome) and *T. theileri* (Fig. 8). Tabanids from Namitangurine presented the highest *T. congolense* infection rate (15.6%), followed by Botao (12.5%) and Zalala (5.6%).

## Discussion

Despite the important role of tabanids and tsetse flies in the transmission of diseases, little research attention has been devoted to these taxa in the Afrotropics, including Mozambique. Limited fundamental information on their classification and distribution patterns are available, with the last major work focused on elucidating these aspects having been conducted more than half a century ago, in Mozambique [24]. Few surveys aiming at the distribution [16], genetic characterization [15] or infection of tsetse flies [65, 66] were previously published.

The results regarding the diversity of tabanids from this study are similar to those of Ahmed et al. [67] in Nigeria, Lendzele et al. [33] in Cameroon, Koné et al. [68] in Burkina Faso and Taioe et al. [23] in South Africa and Zambia. These authors also recorded a greater abundance and diversity of *Tabanus versus* other tabanid genera. *Tabanus par* and *Atylotus agrestis* were the dominant tabanid species in West Africa, despite the large geographical separation, possibly indicative of these species preferring tropical climates. High numbers of *T. par* were also recorded by Taioe et al. [23] in KwaZulu Natal, South Africa, but not *A. agrestis*. KwaZulu Natal is part of the eastern tropical corridor, reinforcing the idea that *T. par* might be a dominant species in tropical Africa. In their studies, Ahmed et al. [67] and Taioe et al. [23] also recorded a low abundance and diversity of *Haematopota*. This is a clear contrast with the Catalogue of Afrotropical Tabanidae by Chainey & Oldroyd [69] that reports approximately 250 *Haematopota* species of a total of 700 tabanid species present in the Afrotropical region. The reason for this contrast is unclear, but possibly the trapping methodology might not be favorable for *Haematopota* species in the region. The complete lack of Chrysopsinae in the trapping is similarly curious and unexpected as it was reported by both Esterhuizen [70] and Taioe et al. [23], both using H traps. It might be possible that species of Chrysopsinae are not easily captured by blue traps, designed for *Glossina* spp., despite their well-known blood-feeding behavior.

*Atylotus* spp. could not be separated confidently and a collapsed group comprising *A. agrestis*, *A. diurnus* and *A. nigromaculatus* was recovered. All specimens in this study were morphologically identified as *A. agrestis*. This requires further study and might reflect incorrectly identified specimens uploaded to GenBank, or the inability of *cox*1 to distinguish between *Atylotus* species. Alternatively, the taxonomy may not correspond to the genetic isolation prescribed by the biological species concept. Cytochrome oxidase *cox*1 barcodes for *Haematopota* species from the Afrotropics are needed. The specimen sequenced in this study (specimen 57) fell sister to a monophyletic group of *H. fenestralis* sequences from a single study. This might reflect confirmation of *H. fenestralis* or a closely related sister species.

All specimens within the Fraternus group as described by Oldroyd [27] were here identified as *T. taeniola*, despite four specimens grouping separately (specimens 18, 53–55), with one specimen identified as *T. fraternus*. Due to a lack of *T. fraternus* reference sequences available and the subtle morphological difference, especially in specimens stored in 70% ethanol (*T. fraternus* has a slight additional pigment on the wings, lacking in *T. taeniola*), confident identification was impossible. This grouping remains an interesting one and the species boundaries are worth investigating. The species boundaries between *T. par* and *T. thoracinus* remains unclear and was not treated in the most recent work by Mugasa et al. [64], who recovered two “genetic variants” from Uganda in their study. Unfortunately, Mugasa et al. [64] did not include *T. par* specimens and could not find morphological differences between the genetic variants. With the sheer abundance of *T. par* in southern and eastern Africa, this species status also deserves investigation.

The performance of *cox*1 as a Tabanidae species delimitating barcode should be reassessed as it posed several problems of which an inadequate number of sequences from accurately identified specimens. Other molecular markers, such as the internal spacers in the rRNA gene (ITS1 and ITS2) are worth investigating as an alternative for species delimitation. It should be noted, however, that *cox*1 worked well considering *Glossina*, but *Glossina* is considerably less diverse and all species have several representative sequences on GenBank. The fact that no sequences from *G. pallidipes* were obtained may be due to the poor quality of the PCR product sent for sequencing.

The underlying reasons for the observed differences in tsetse and tabanids abundance between the selected sites are unclear. Vegetation type, vegetation cover and non-domesticated host species (host-preference), have all been shown to be important variables in the distribution and composition of haematophagous fly populations [71–73]. Zalala is an area consisting of small and widely dispersed pockets of shrubs and trees that may provide insufficient habitat for a high diversity of flies. Moreover, cattle being the only apparent food source available in the area is an additional limiting factor to the expansion of both tabanid- and tsetse fly assemblages. Finally, the dense human settlements that are rapidly expanding and changing the surrounding environment may in turn also negatively impact biodiversity. Haematophagous flies that possibly require a wide range of hosts may then be especially affected [74–78].

Namitangurine on the other hand, is characterized by dense thickets and is seemingly a less disturbed area with wild animals like sable, kudu, reedbuck, duiker and bush pig present [79]. Interestingly, the highest infection rates were also detected from this area, possibly reflecting the importance of wild animals as reservoirs of *Trypanosoma* species. In fact, salivarian trypanosomes can be found in a wide variety of hosts such as ruminants, carnivores, rodents and reptiles [80–83]. This might then indicate the important role of these animals for the circulation of trypanosomes in a certain habitat. Control measures directed to trypanosomes reservoirs have shown to be successful in the reduction of trypanosomosis prevalence, though they have proven not to be environmentally sustainable [84–86].

The efficacy of blue traps for determining Tabanidae diversity should perhaps be revisited, with all aforementioned studies being unable to reflect the “true” diversity of Tabanidae species as reported from museum data [25–27, 87]. Furthermore, the higher diversity recorded in regions from museum data might possibly reflect “false” diversity due to erroneous taxonomy and highlight the need for renewed taxonomic approaches as stipulated by Morital et al. [18].

It is important to note that the H trap had a higher efficacy in capturing *G. brevipalpis*, compared to both the Epsilon- and NGU traps. However, Malele et al. [88] reported that, in Tanzania, H traps did not capture any G. *brevipalpis*. Several other studies, in turn, have demonstrated good performance of H traps in the capture of *G. brevipalpis* in South Africa [34, 89, 90] and in Mozambique [16]. These contradicting results are important in control and surveillance studies and require clarification.

The low levels of Shannonʼs diversity index obtained in the present study were mainly due to the high frequency of *T. par* (66.8%) and *Atylotus agrestis* (23.2%), showing the existence of clear dominant species and a very low evenness of species. This is often found in areas where human disturbances are evident, and cattle grazing might form part of such a disturbance [91]. The rainy season accounted for two folds the total capture number of the dry season as recorded by several studies [28, 33, 67, 92]. On a finer scale, seasonal tabanid activity peaks is extremely important to understand the pathogen transmission dynamics. In this region of Mozambique, an early wet-season peak was observed in November, followed by a second more pronounced peak in March, at the end of the rainy season. This might be bivoltine behavior, with the emergence of the dry-season generation that probably included diapause (first peak), and a subsequent wet-season generation that lacks diapause (second peak). Investigating the drivers of these peaks might prove valuable for predicting, mitigating and controlling diseases spread by horse flies [93].

In this study, *G. brevipalpis* appears to be the most important vector since the species was most often collected and showed a high infection rate (72.2%, *n* = 18). Motloang et al. [94] reported contrasting results from an experimental study in South Africa; the authors concluded that, despite their higher abundance, the role of *G. brevipalpis* in the transmission of *T. congolense* was negligible. The results here, in turn, warrants additional investigation and to reassess the role of *G. brevipalpis* in the transmission of trypanosomosis. Over the course of a year’s uninterrupted collection, *G. brevipalpis* was the only species present in all three study areas with individuals from all three areas infected with *Trypanosoma*.

Although the number of captured *G. morsitans* flies was low (four individuals), their high infection rate (100%) could nevertheless indicate their important role as a vector of trypanosomosis in this area. *Glossina morsitans* was described by Vreysen et al. [7] as one of the most important species in the Morsitans group and the major vector of AAT in eastern and southern Africa, and it was experimentally proven by Reinfeberg et al. [95] that Morsitans flies were more susceptible to *T. congolense* infections compared to other groups, as Palpalis for example. Thus, the high infection rate found in G*. morsitans* from Namitangurine is somewhat expected. Even though infection rates differ from those found in this study, this is congruent with the findings of Salekwa et al. [96] in Tanzania and Shereni et al. [97] in Zimbabwe. These studies postulated that *G. morsitans* from conservation or less disturbed areas (like Namitangurine) are more likely to be infected with *Trypanosoma* species.

Since only one *G. pallidipes* specimen was captured, no accurate discussion can be presented on the infection rate of this species. However, in other studies, *Trypanosoma* infection were detected in *G. pallidipes* [31, 98], which shows that they can actively harbor and transmit trypanosomes. The presence/absence of infection in tsetse flies can be due to feeding preferences [99], genetic differences, the availability of reservoirs, the parasitaemia of the vertebrate host [100, 101] and the nutritional status of the flies [102, 103]. As discussed previously, tsetse flies respond demographically to habitat destruction or fragmentation. However, it is known that the flies also respond physiologically to this process and to other ecological pressures. In fact, it was experimentally proven that environmental stress, including starvation, causes increase in the susceptibility of tsetse flies to trypanosome infections [102–105]. This may explain the high infection rates detected in the present study despite the small sample size.

The only pathogenic trypanosome found in the present study was *T. congolense*. This is well in accordance with the study of Mulandane et al. [22] reporting *T. congolense* as the only circulating pathogenic *Trypanosoma* species in cattle in Nicoadala District. Additional to *T. congolense*, *T. theileri* was also found in tabanids in all the species screened except for *A. africana*, which is not an indication that this species cannot harbor trypanosomes as Firmino et al. [106] detected trypanosome sequences in *A. africana* flies captured in Ethiopia. *Trypanosoma theileri* is a stercorarian, non-pathogenic trypanosome transmitted to cattle by tabanids, where it undergoes a developmental cycle [107, 108], and highly prevalent around the globe [109–112].

Our results strongly suggest a role for tabanids in the transmission of *T. congolense* in the study area. In general, the participation of tabanids in the dynamics of African animal trypanosomosis (linked to *T. vivax* and *T. evansi*) is rightly becoming more important with the ecological transformations that are progressively affecting the tsetse densities and distribution in Africa [113–115]. Moreover, mechanical transmission of trypanosomosis, depending on the circumstances and conditions, may be as efficient as biological transmission, although its potential impact has never been estimated [8, 9, 19]. These inferences can be supported by the findings from Desquesnes & Dia [113, 116], where mechanical transmission of *T. congolense* and *T. vivax* by *A. agrestis* and *A. fuscipes* were experimentally demonstrated. They are also supported by Abebe & Jobre [117], in which high *T. vivax* and *T. congolense* infection where detected in tsetse free zones. Our study indicates that *A. agrestis* could have an important impact on the dynamics and/or epidemiology of trypanosomosis.

## Conclusions

To the best of our knowledge, this is the first study conducted in Nicoadala District, an area identified as a drug resistance hotspot, involving the characterization of the Tabanidae and Glossinidae and the detection of the trypanosomes they harbor. As a pioneer study, it constitutes a starting point for future work on the subject. Considering the trapping effort, it can be concluded that a very small population of tsetse flies responsible for the biological transmission of trypanosomosis is present in the district. However, the strong presence of tabanids, including species that have previously been identified as trypanosomosis vectors, suggests their participation as mechanical vectors as a relay and amplification factor to tsetse flies resulting in a high prevalence of trypanosomosis in the district as reported by Mulandane et al. [22], Jamal et al. [118] and Specht [119].

## Supplementary information

**Additional file 1: Figure S1.** An uncorrected p-distance data-display network, using all characters recovered from SplitsTree using the Tabanidae *cox*1 data. Bootstrap (bs) support calculated from 1000 replicates is indicated for the various groupings. The double-digit numbers (intuitional voucher numbers) on the tips of the network represent the sequences from this study.

**Additional file 2: Figure S2.** An uncorrected p-distance data-display network, using all characters recovered from SplitsTree using the Glossinidae *cox*1 data. Bootstrap (bs) support calculated from 1000 replicates is indicated for the various groupings. The double-digit numbers on the tips of the network represent the sequences from this study. Red arrows indicate the specimen sequences from this study.

**Additional file 3: Table S1.** Metadata for all specimens analysed.

## Data Availability

Captured flies and DNA extracted in this study are deposited at the Biotechnology Center Laboratory (Eduardo Mondlane University) in Maputo, Mozambique. The newly generated sequences were submitted to the GenBank database under the Accession numbers MT231157–MT231193.

## Abbreviations

PCR: polymerase chain reaction
H trap: horizontal trap
ddH_2_O: double-distilled water
rRNA: ribosomal ribonucleic acid
ANOVA: analysis of variance
*SSU*-rDNA: small subunit of ribosomal deoxyribonucleic acid
ITS: internal transcribed spacer
