# Supplementary figures and images for: Evaluation of the relative roles of the Tabanidae and Glossinidae in the transmission of trypanosomosis in drug resistance hotspots in Mozambique
Source: Parasit Vectors. 2020 Apr 29;13:219. doi: 10.1186/s13071-020-04087-1 (PMC7189697; doi:10.1186/s13071-020-04087-1)

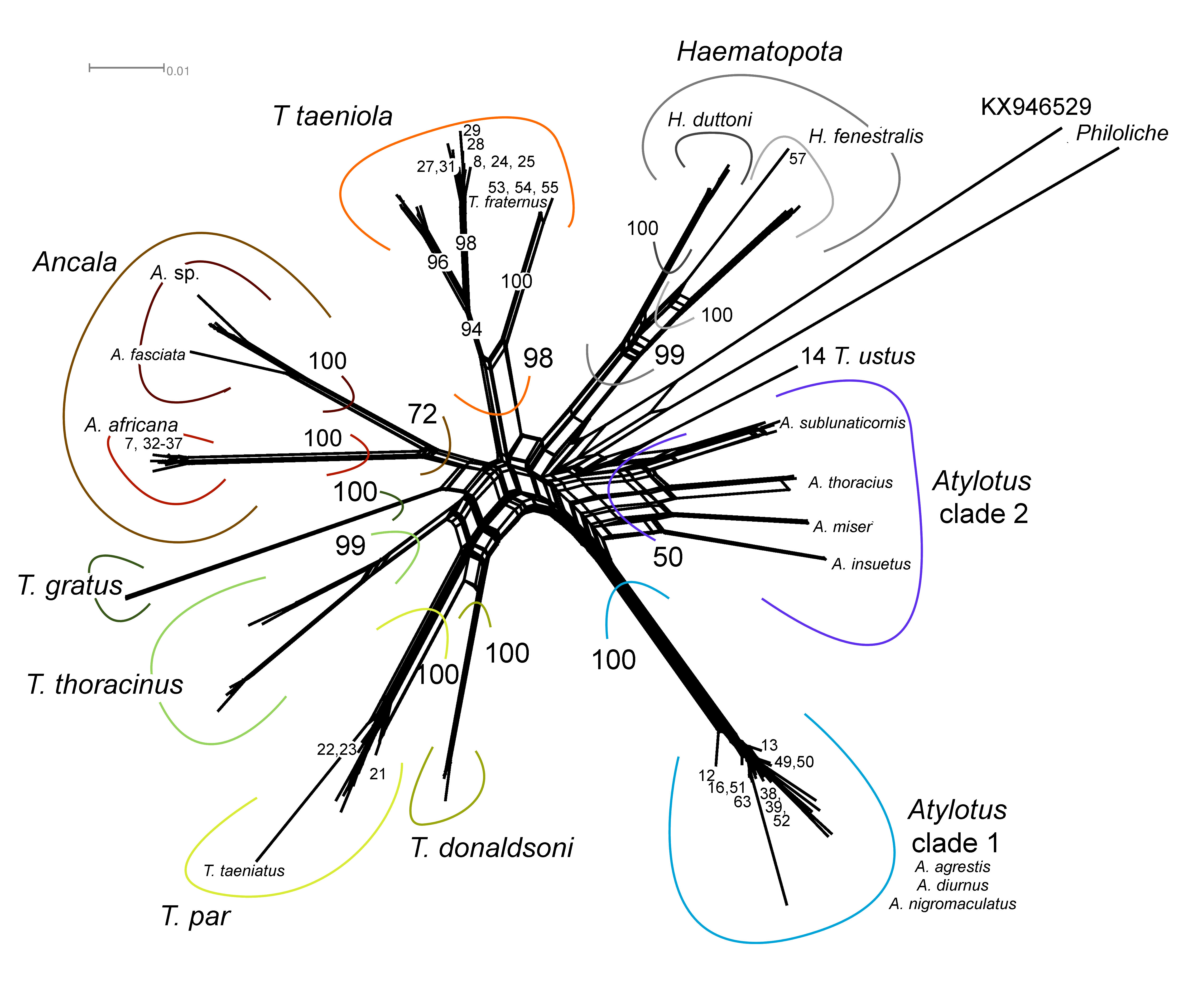

Supplement: Supplementary file 1 — Additional file 1: Figure S1. An uncorrected p-distance data-display network, using all characters recovered from SplitsTree using the Tabanidae cox1 data. Bootstrap (bs) support calculated from 1000 replicates is indicated for the various groupings. The double-digit numbers (intuitional voucher numbers) on the tips of the network represent the sequences from this study. [file 13071_2020_4087_MOESM1_ESM.jpg]

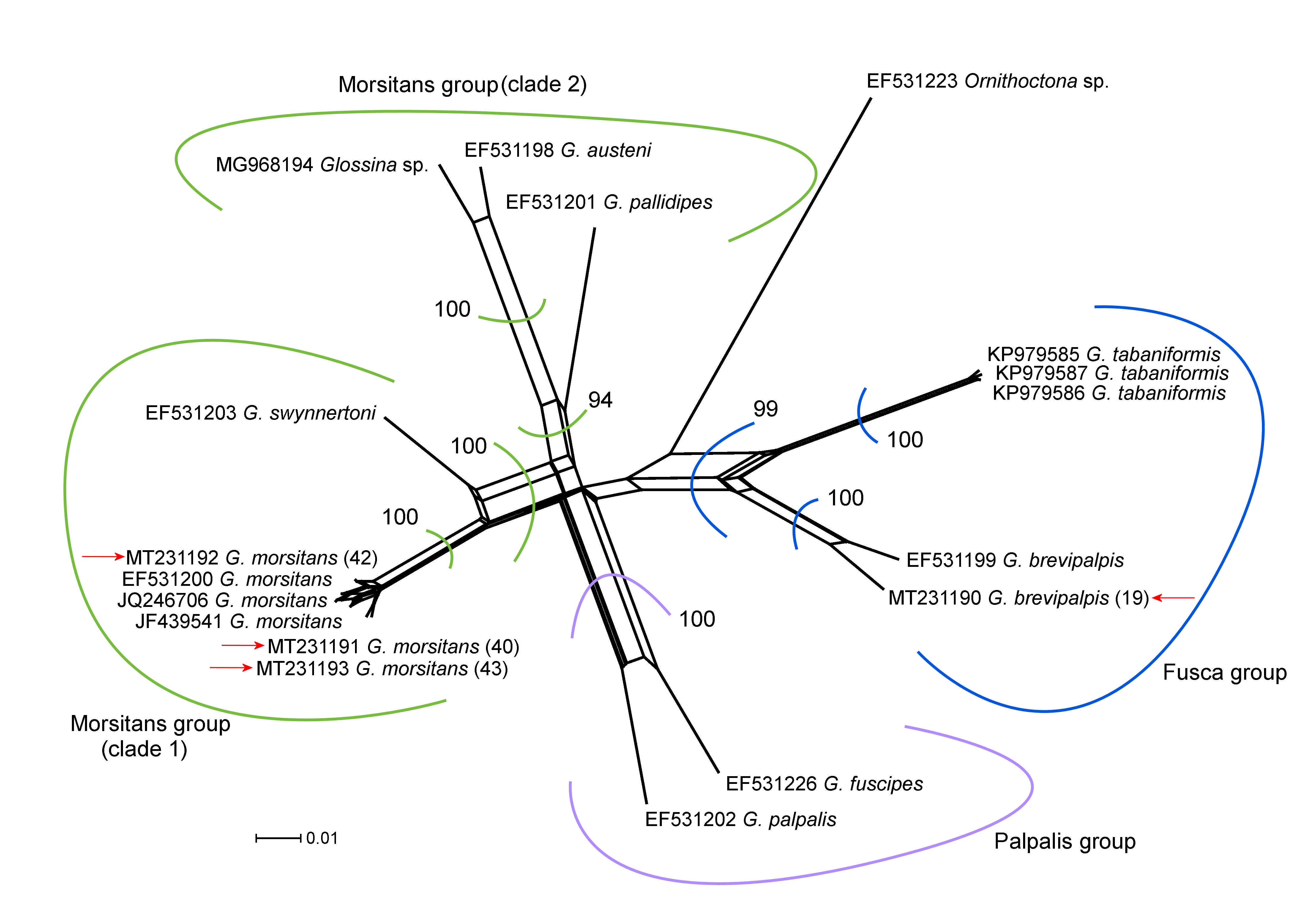

Supplement: Supplementary file 2 — Additional file 2: Figure S2. An uncorrected p-distance data-display network, using all characters recovered from SplitsTree using the Glossinidae cox1 data. Bootstrap (bs) support calculated from 1000 replicates is indicated for the various groupings. The double-digit numbers on the tips of the network represent the sequences from this study. Red arrows indicate the specimen sequences from this study. [file 13071_2020_4087_MOESM2_ESM.jpg]
